# The Proanthocyanidin-Rich Fraction Obtained from Red Rice Germ and Bran Extract Induces HepG2 Hepatocellular Carcinoma Cell Apoptosis

**DOI:** 10.3390/molecules24040813

**Published:** 2019-02-23

**Authors:** Supranee Upanan, Supachai Yodkeeree, Pilaiporn Thippraphan, Wanisa Punfa, Rawiwan Wongpoomchai, Pornngarm Limtrakul (Dejkriengkraikul)

**Affiliations:** 1 Anticarcinogenesis and Apoptosis Research Cluster, Faculty of Medicine, Chiang Mai University, Chiang Mai 50200, Thailand; supranee_molecular@hotmail.com (S.U.); yodkeelee@hotmail.com (S.Y.); wanisapun@hotmail.com (W.P.); rawiwan.wong@cmu.ac.th (R.W.); 2 Department of Biochemistry, Faculty of Medicine, Chiang Mai University, Chiang Mai 50200, Thailand; tipprapant@gmail.com; 3 Center for Research and Development of Natural Products for Health, Chiang Mai University, Chiang Mai 50200, Thailand

**Keywords:** proanthocyanidin, red rice germ and bran extract, apoptosis, hepatocellular carcinoma

## Abstract

This study aims to determine the anti-carcinogenic effects of the proanthocyanidin-rich fraction (PRFR) obtained from red rice germ and bran extract on HepG2 cells. The PRFR obtained from red rice germ and bran extract could reduce the cell viability of HepG2 cells as shown by the IC_50_ value at 20 µg/mL. Notably, PRFR concentrations at 20 and 40 µg/mL significantly increased the number of cells in the G2/M phase from 25.7% ± 1.4% in the control group to 36.2% ± 3.4% (*p* < 0.01) and 48.9% ± 2.6% (*p* < 0.0001), respectively, suggesting that the cells were arrested in this phase, which was confirmed by the reduction of survival proteins, including cyclin B1 and cdc25. Moreover, the PRFR at 20 and 40 µg/mL could induce cell death via the apoptosis cascade, indicated by the percentage of total apoptotic cells from 9.9% ± 3.1% in the control group to 41.1 ± 3.9 (*p* < 0.0001) and 82.2% ± 5.8% (*p* < 0.0001), respectively. This was clarified by increasing apoptotic proteins (such as cleaved PARP-1, cleaved caspase-8 and cleaved caspase-3) and decreasing anti-apoptotic protein survivin without p53 alterations. These results demonstrated that the PRFR obtained from red rice germ and bran extract could inhibit cell proliferation and induce cell apoptosis in HepG2 cells via survivin, which could potentially serve as a new target for cancer therapeutics making it an excellent “lead candidate” molecule for in vivo proof-of concept studies.

## 1. Introduction

Nowadays, several types of pigmented rices such as red rice, purple rice and black rice have become popular as functional foods compared to white rice due to the high contents of valuable phytochemicals found in their pigments. Previously reported data have verified that pigmented rice contains higher amounts of phenolic and flavonoid compounds, such as anthocyanins and proanthocyanidin, than white rice [1]. Interestingly, anthocyanins in black rice and proanthocyanidin in red rice have been widely studied as active ingredients that possess a variety of advantages, including anti-oxidant, anti-inflammation and anti-carcinogenesis properties [1,2,3,4,5,6].

Proanthocyanidin, well known condensed tannins, are a class of polymeric phenolic compounds primarily consisting of flavon-3-ol units—catechin, epicatechin, 3-*O*-gallates and epigallates—that can be found in high concentrations in many sources, especially in grape seeds and red rice [1,3,4,6,7,8,9,10]. Our previous studies have demonstrated that proanthocyanidin derived from red rice extract displayed a notable degree of potency as anti-cancer, anti-inflammatory and anti-oxidant agents. Initially, we found that red rice grain extracts exhibited anti-invasive activity in certain cancer cell lines (e.g., human breast carcinoma, MDA-MB-231 and human fibrosarcoma, HT1080) [3]. Next, we showed that the proanthocyanidin-rich fraction derived from red rice grain extracts could inhibit a metastasis cascade by affecting MMP, uPA, IL-6 and ICAM-1 in the breast cancer cell line, MDA-MB-231 [4]. With regard to the potential inflammatory effects, red rice grain extract could reduce UVB-induced inflammation and aging in primary human skin fibroblasts via the inhibition of the MAPK signaling pathway by decreasing NF-κB and AP-1 activation through a reduction in IL-6 and IL-8 production leading to a decline in ECM degradation (i.e., MMP-1 expression and MMP-2 and collagenase activity) and an increase in ECM synthesis (i.e., collagen and hyaluronic acid synthesis) [11]. Moreover, in LPS-treated Raw 264.7 macrophage cells, red rice grain extract could suppress the production of IL-6, TNF-α, NO, iNOS and COX-2, inhibiting the MAPK signaling pathway by reducing NF-κB and AP-1 activation [5]. Consistently, the proanthocyanidin- rich fraction obtained from red rice grain extracts exhibited anti-oxidant and anti-aging properties in primary human skin fibroblasts [12]. Therefore, proanthocyanidin derived from red rice could be potent natural products serving as an alternative option for cancer chemotherapy.

However, the anti-carcinogenesis properties of red rice proanthocyanidin against liver cancer or hepatocellular carcinoma has not yet been clarified. Currently, primary liver cancer is amongst the commonest tumors worldwide, particularly in parts of the developing countries including Thailand, and is increasing in incidence [13,14,15]. Hepatocellular carcinoma (HCC), the most frequent primary liver cancer, has been reported as the sixth most common cancer in worldwide, the second cancer death cause in men and the sixth in women [16]. HCC is caused by liver cirrhosis resulting from either viral hepatitis (i.e., hepatis B and C virus) or non-viral chronic liver diseases (e.g., alcohol, fatty acid, autoimmune or genetic metabolic liver diseases) [17,18]. HCC is usually treated by medical (e.g., chemo-radiation and sorafenib) and surgical treatments (i.e., liver resection or liver transplantation) [13,16,18]. The treatment for liver cancer depends on the stage of the condition. Patients with early stage tumors that can be removed surgically have the best chance of long-term survival, with 5 year survival rates ranging up to 30% to 50% [13]. Unfortunately, primary liver cancer is rarely detectable early, when it is most treatable, secondary or metastatic liver cancer is typically too advanced to permit surgery and it is hard to treat, hence its much lower survival rate. In some patients, chemotherapy is administered directly into the liver (chemoembolization) to reduce tumors to a size that may make surgery possible. Choosing the correct chemotherapy including the type and duration is very important for the efficiency of the cancer treatment. Some patients require multiple drug treatments to reduce the severe side effects if one drug with high doses has been applied [13,16,18]. Therefore, the effective liver cancer treatment reducing its side effects is challenging. Here, it is an issue of significant interest for the preliminarily study of the anti-carcinogenic effects of the proanthocyanidin-rich fraction (PRER) derived from red rice germ and bran extract on hepatocellular carcinoma, HepG2 cells.

## 2. Results

### 2.1. Effect of PRFR on Hepatocellular Carcinoma cell Viability 

**Figure 1:**
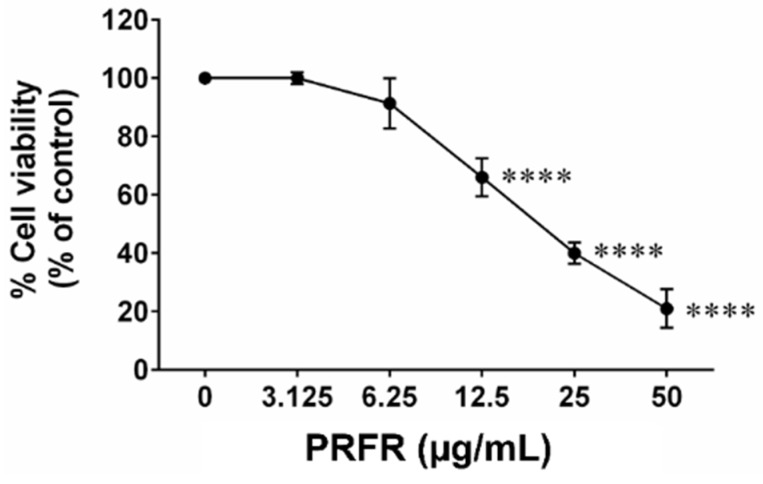
Effect of PRFR on the HepG2 cells viability. The cells were treated with or without various concentrations (0–50 µg/mL) of PRFR for 48 h. The cell viability was determined using SRB assay. All assays were demonstrated in triplicate in three independent experiments and the mean ± standard deviations are shown. **** *p* < 0.0001 versus the control.

The % yield of PRFR from red rice germ and bran was 0.23% (*w*/*w*). The total amount of proanthocyanidin in the PRFR was 177.22 ± 16.66 mg catechin/g extract. We firstly determined the cytotoxicity of PRFR in HepG2 cells using the SRB assay. PRFR dramatically decreased the viability of HepG2 cells after being treated with 0–200 µg/mL for 48 h. The PRFR exhibited toxicity against HepG2 with IC_50_ at approximately 20 μg/mL (Figure 1).

### 2.2. Effect of PRFR on G2/M cell Cycle Arrest in HepG2 Cells

**Figure 2:**
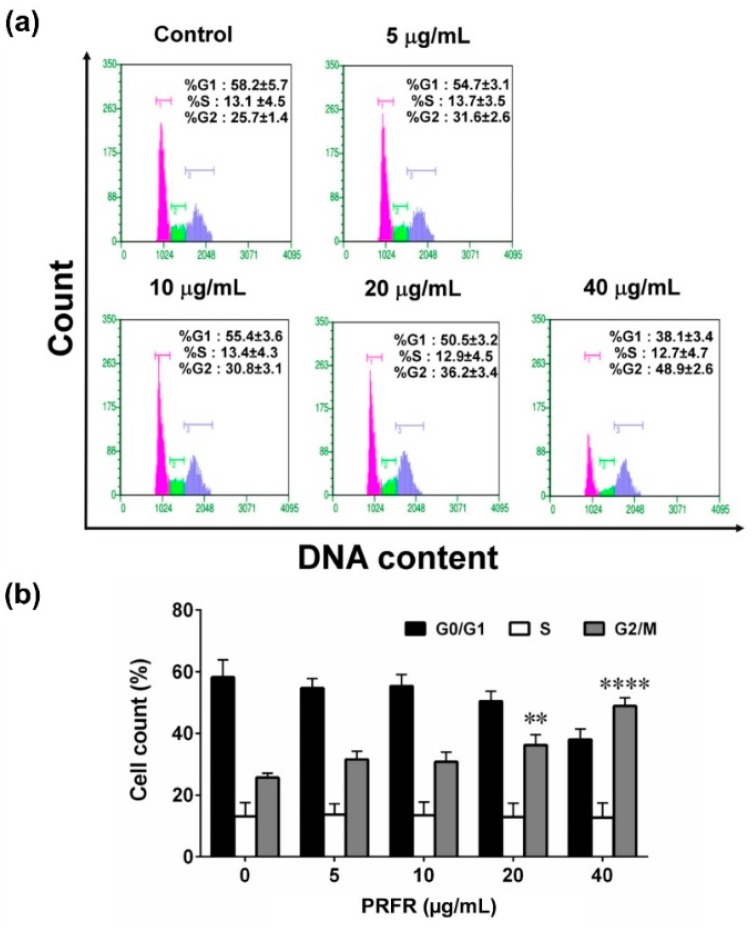
Effect of PRFR on HepG2 cells cycle arrest. The cells were incubated with or without various concentrations (0–40 µg/mL) of PRFR for 48 h. Cell cycle arrest was determined using Guava Cell cycle analysis (**a**). All assays were performed in triplicate and the mean ± standard deviations are shown as the histogram (**b**). ** *p* < 0.01 and **** *p* < 0.0001 versus the control.

Cell cycle arrest was determined using Guava Cell cycle analysis. After treating HepG2 cells with or without PRFR at various concentrations (0–40 µg/mL) for 48 h, the cells tended to arrest in the G2/M phase when compared to the non-treated cells (Figure 2a). At 20 and 40 µg/mL of PRFR, the percentage of the cells in the G2/M phase was significantly increased from 25.7% ± 1.4% in the control group to 36.2% ± 3.4% (*p* < 0.01) and 48.9% ± 2.6% (*p* < 0.0001), respectively (Figure 2b), suggesting that PRFR could inhibit cell proliferation by arresting cells in the G2/M phase.

### 2.3. Effect of PRFR on cell Cycle Regulated Protein Expression in HepG2 Cells

**Figure 3:**
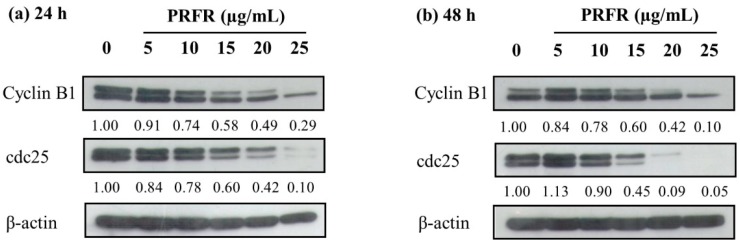
Effect of PRFR on survival protein expression in HepG2 cells. The cells were incubated with or without PRFR (0–25 µg/mL) for 24 h (**a**) and 48 h (**b**). The expression of survival proteins regulating the cell cycle was detected by western blot analysis. The band intensity has been shown as a relative ratio of the interested protein to β-actin. Data from a typical experiment are depicted here and similar results were obtained in three independent experiments.

To investigate the molecular mechanism of PRFR in the regulation of G2/M cell cycle arrest, the expression level of the cell cycle regulated proteins was evaluated using western blot analysis. Cyclin B1 and cdc25 proteins are the potential candidates of the proteins involved in cell proliferation in cancer cells by inducing cell cycle progression. As shown in Figure 3, the treatments with 0–25 µg/mL of PRFR clearly reduced the expression levels of cyclin B1 and cdc25 in a dose-dependent manner at incubation times of both 24 h and 48 h. The results demonstrate that the reduction in cell proliferation, due to the PRFR treatment, resulted from decreases in cyclin B1 and cdc25 protein in arresting the cells at the G2/M phase. 

### 2.4. Effect of PRFR on HepG2 cell Apoptosis

**Figure 4:**
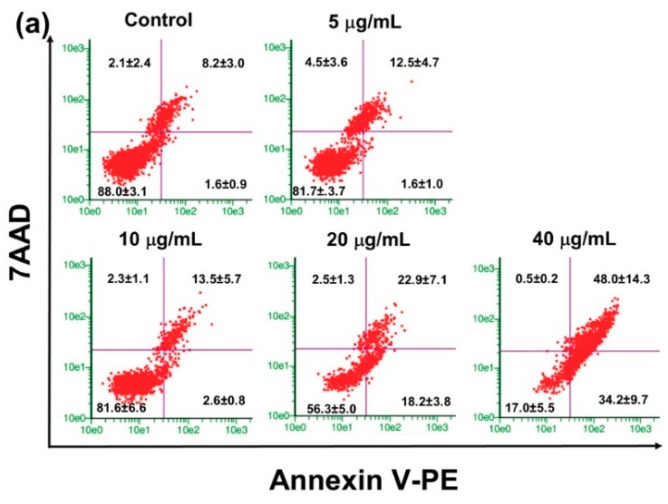

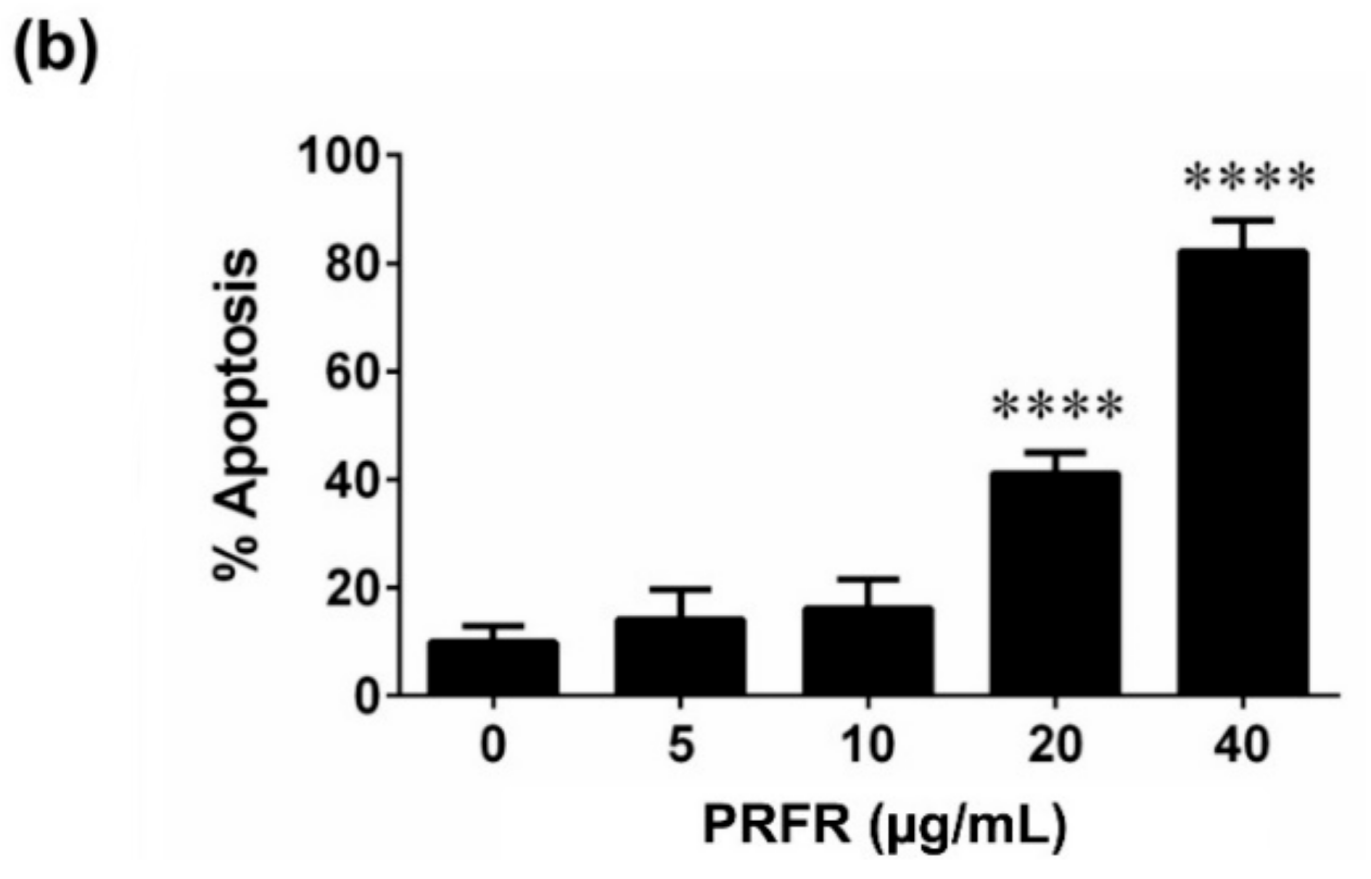
Effect of PRFR on HepG2 cells apoptosis. The cells were incubated with or without PRFR (0–40 µg/mL) for 48 h. Cell apoptosis was determined using Guava Nexin analysis (**a**) Annexin V-PE positive cells indicated early apoptosis, while double positive cells indicated late apoptosis. The percentages in early and late apoptosis were summed up; all assays were performed in triplicate in three independent experiments and the mean ± standard deviations are shown as the histogram (**b**). **** *p* < 0.0001 versus the control.

The anti-proliferative effect of PRFR on HepG2 cells was determined using Guava Nexin analysis. HepG2 cells were treated with PRFR (0–40 µg/mL) for 48 h and stained with annexin V-PE and 7AAD. PRFR could elevate the population of (early and late) apoptotic HepG2 cells in a dose dependent manner (Figure 4a). PRFR at dosages of 20 and 40 µg/mL of PRFR could significantly increase the percentage of total apoptotic cells from 9.9% ± 3.1 in the control group to 41.1 ± 3.9 (*p* < 0.0001) and 82.2% ± 5.8% (*p* < 0.0001), respectively (Figure 4b). Thus, the data suggested that PRFR exhibited anti-proliferation properties in HepG2 cells by stimulating cell apoptosis. 

### 2.5. Effect of PRFR on Apoptotic Proteins, Anti-Apoptotic Protein Survivin and Tumor Suppressor Protein p53 in HepG2 Cells

**Figure 5:**
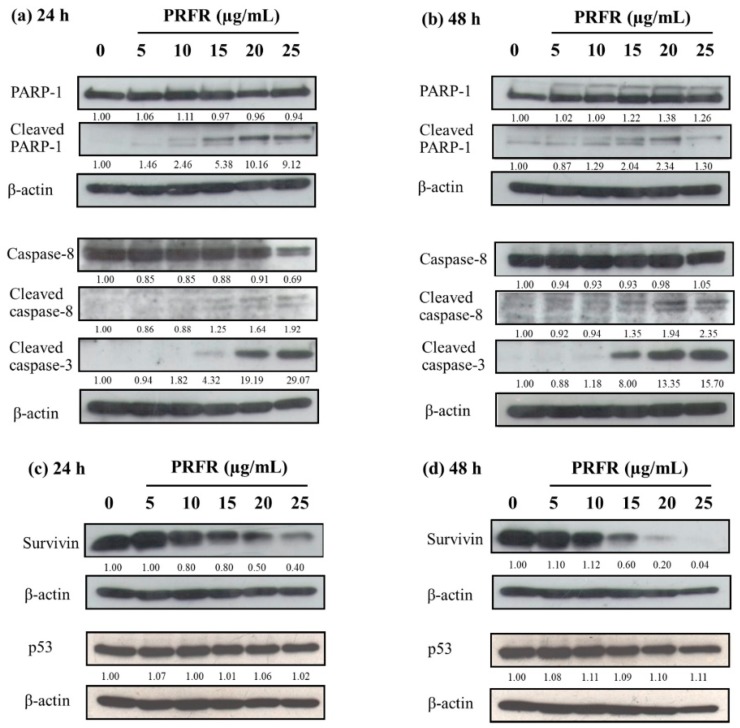
Effect of PRFR on apoptotic proteins, anti-apoptotic protein survivin and tumor suppressor protein p53 in HepG2 cells. The cells were incubated with or without PRFR (0–25 µg/mL) for 24 h (**a**,**c**) and 48 h (**b**,**d**). The expression of proteins was detected by western blot analysis. The band intensity has been shown as a relative ratio of the interested protein to β-actin. Data from a typical experiment are depicted here, while similar results were obtained in three independent experiments.

Consistent with western blot analysis after PRFR treatment (0–25 µg/mL), the protein levels of cleaved PARP-1, cleaved caspase-8 and cleaved caspase-3 were dramatically increased as the active form of the apoptotic cascade in a dose and time response manner at incubation times of 24 h and 48 h (Figure 5a,b). 

Interestingly, the survivin protein level was also greatly reduced in a dose and time response manner at 24 h and 48 h without alteration of the p53 protein level (Figure 5c,d). The results revealed that the PRFR could induce apoptosis by enhancing active apoptotic proteins and by dropping the anti-apoptotic protein, survivin, with no p53 alteration.

## 3. Discussion

In accordance with the current trend toward healthy foods, people are becoming more likely to consume pigmented rice (e.g., red and black rice) than white rice. Several research studies have reported on the benefits of red rice by highlighting its potential anti-oxidative, anti-inflammatory and anti-cancer properties [1,3,4,5,6]. There have been reports that demonstrate that red rice contains high levels of flavonoids and phenolic compounds, particularly proanthocyanidin [1,3,6]. Proantho-cyanidin in plants (e.g., grape seeds and blackberries) have been investigated as potential anti-carcinogenic, anti-inflammatory and anti-oxidant agents [7,8,9,19]. In addition, our previous studies have demonstrated that red rice grain extract contains a high proanthocyanidin content and displays an anti-metastasis effects on human breast carcinoma, MDA-MB-231 cells and human fibrosarcoma, HT1080 cells [3], while also displaying an anti-inflammatory effect on LPS-treated Raw 264.7 macrophage cells [5] and UVB-induced inflammation primary human skin fibroblast cells [11]. PRFR was then prepared from red rice germ and bran extract using column chromatography. The total proanthocyanidin content was estimated by a vanillin assay; the proanthocyanidin profile was qualified by HPLC chromatography, showing gallocatechin (GC), epigallocatechin (EGC), catechin (C) and epicatechin (EC) peaks [4]. Interestingly, we clarified that PRFR prepared from red rice grain extract, which was dehulled without removing germ and bran, still exhibited its anti-cancer invasive properties in human breast cancer, MDA-MB-231 cells [4] and revealed anti-oxidant capacity in primary human skin fibroblasts [12]. We hypothesized that the germ and bran of whole grained rice are the primary sources of beneficial phytochemicals including proanthocyanidin. Next, the present study primarily sought out the benefits of PRFR derived from red rice germ and bran extract as a potential anti-cancer agent in the treatment of liver cancer. The results showed that PRFR derived from red rice germ and bran extract could inhibit human hepatocellular carcinoma, HepG2 cell proliferation via the induction of cell arrest in the G2/M of the cell cycle. As a result of the PRFR treatment, the down-regulation of cyclin B1 and cdc25 proteins, a marker of cell cycle progression in the G2/M phase, forced the cells to stop at the G2/M phase. Cyclin B1-CDK1 complex is known as a promoting factor driving G2/M progression; the activation by dephosphorylating CDK1 by phosphatase cdc25 is recognized as a rate-limiting step during G2/M transition [20,21]. Therefore, the HepG2 cells could not pass the G2/M phase and stopped growing due to a reduction in cyclin B1 and cdc25 proteins caused by the PRFR treatment.

Furthermore, in HepG2 cells, the PRFR not only inhibited cell proliferation, but also induced cell death via the apoptosis cascade. After treatment with PRFR, the number of apoptotic cells increased in both early and late apoptosis. In addition, the PRFR activated PARP-1, caspase-8 and caspase-3 cleavage, indicating that it could enhance apoptosis in HepG2 cells. Apoptotic proteins (e.g., PARP-1, caspase-8 and caspase-3) and anti-apoptotic proteins (e.g., survivin) were involved in apoptosis pathway [22,23,24]. PARP-1 is a nuclear enzyme involved in DNA repair, DNA stability, and transcriptional regulation; it acts as a characteristic event of apoptosis [24]. Anti-apoptotic protein survivin is normally suppressed by tumor suppressor protein p53 by binding to its promotor. Notably, it is overexpressed in various cancer cells [25,26,27]. However, there have been reports that have found no association between survivin and p53 protein expression in the liver, colorectal and lung tumors of Thai cancer patients. Additionally, survivin protein could be detected in 88.9% of the cases, whereas p53 protein was accumulated in 52.7% of those cases [28]. This suggested that survivin overexpression might not totally depend on p53 aberration. Similarly, survivin down-regulation that resulted from the PRFR treatment may not be associated with p53 expression. Nevertheless, the method to effectively down-regulate survivin would be one of the optimal targets for cancer therapy among Thai patients. Interestingly, survivin also regulates the transition of G2/M phase in the cell cycle [29], relating to the discussion above. In the caspase pathway, survivin also blocks apoptosis by binding caspases and inhibiting the caspase activity and cell death [30]. The active form of cleaved caspase-8 activates caspase-3 by cleaving caspase-3 to an active form; the activated caspase-3 then cleaves its substrates to process further apoptotic cell death [22,23]. Thus, the reduction of survivin, resulting from the PRFR treatment, would induce HepG2 cell arrest in the G2/M of the cell cycle as well and elevate caspase activity for processing the cleavage into active caspases, finally leading to cell death via apoptosis pathway.

Recently it has been established that diverse beneficial herbs may also have poisonous side effects. Therefore, toxicity assessments for natural product have been a concern. The in vivo safety and toxicity studies of proanthocyanidin have reported that the lethal dose 50 (LD_50_) of proanthocyanidin from grape seed extract is higher than 5000 mg/kg in rat [31] and no adverse effect was found in a subchronic toxicity study with 1500 mg/kg /day of proanthocyanidin in rats [32]. We have previous reported that proanthocyanidin from red rice are the oligomeric with the same degree of polymerization as proanthocyanidin found in grape seed extract [4]. Clinical data relating to the safety of proanthocyanidin extract were available from an open-label study in healthy Japanese male and female volunteers aged 20–64 years. The results shown that oral intake of proanthocyanidin-rich grape seed extracts up to 2500 mg for 4 weeks was found to be generally safe with no significant adverse events, abnormal physical signs, or abnormal biological measurements during the study [33]. Therefore, intake high dose of proanthocyanidin should be considered safe in humans.

Based on our findings and previous data, proanthocyanidin is considered as a potent anti-cancer molecules by regulating numerous molecular mechanisms, and it have the potential to be good therapeutic molecules in the treatment of cancer. Nevertheless, there are a few publications assessing the clinical efficacy of proanthocyanidin as chemotherapeutics in human. The dose of grape seed proanthocyanidin in a clinical study of breast cancer patients was 100 mg three time per day [34]. Therefore, we suggest further investigation of the efficacy of proanthocyanidin as anti-cancer agents in patients with the liver cancer by conducted as a randomized and double-blind study with doses of approximately 100 mg three times per day.

## 4. Materials and Methods

### 4.1. Chemicals and Reagents

Dulbecco’s Modified Eagle Medium (DMEM), trypsin and penicillin-streptomycin were supplied by Gibco BRL Company (Waltham, MA, USA). Anti-β actin antibody and Sulforhodamine (SRB) dye were obtained from Sigma-Aldrich (St. Louis, MO, USA). Antibodies for cyclin B1, cdc25, caspase-8 and cleaved caspase-8, caspase-3 and cleaved caspase-3 and survivin were purchased from Cell Signaling Technology (Boston, MA, USA). Antibody for p53 was supplied by Merck (Darmatadt, Germany). Antibodies for PARP-1 and cleaved PARP-1were obtained from Santa Cruz Biotechnology (Dallas, TX, USA). Fetal bovine serum (FBS), RIPA buffer, protease inhibitors and Coomassie Plus™ Protein Assay Reagent were obtained from Thermo Scientific Company (Waltham, MA, USA). Guava Cell Cycle Reagent and Guava Cell Nexin Reagent were purchased from Merck-Millipore (Darmatadt, Germany). Can Get Signal^®^ Immunoreaction Enhancer Solution was purchased from Toyobo (Osaka, Japan). Nitrocellulose membrane and ECL reagent were supplied from GE Healthcare (Chicago, IL, USA).

### 4.2. Preparation of Proanthocyanidin Rich Fraction (PRFP) Derived from Red rice Germ and Bran 

The red rice (*Oryza sativa* L.) was harvested from the Doi Saket District in Chiang Mai, Thailand. A voucher specimen number was certified by the herbarium at the Flora of Thailand, Faculty of Pharmacy, Chiang Mai University (voucher specimen no. 023148) and stored for future reference. To obtain red rice germ and bran, whole grains of red rice (1 kg) were dehulled and polished using a rice de-husker and a rice milling machine to obtain the rice germ and bran.

Red rice germ and bran extract was prepared by following the previously reported protocol [5]. Briefly, red rice germ and bran (440 g) was soaked in 50% ethanol for 24 h. After 24 h, the mixture was then filtered through filter paper to separate the residue. The filtrated samples were evaporated by a rotary vacuum evaporator (Büchi, Flawil, Switzerland) to obtain concentrated ethanolic fractions. The fractions were further partitioned with saturated butanol using a separating funnel. The upper layer in the butanol fraction was collected to obtain a medium polar fraction. The medium polar fraction was then evaporated to remove the butanol and it was freeze-dried to remove any water to finally obtain red rice bran extract powder.

Proanthocyanidin rich fraction (PRFR) was prepared from the red rice germ and bran extract powder described above using Sephadex LH20 (GE Healthcare) chromatography by a modified method [4]. Briefly, red rice germ and bran extract powder (3.5 g) was dissolved in methanol (10 mL) and loaded onto a Sephadex LH-20 column. The fractions were sequentially eluted with solutions of 70% methanol, 30% methanol and 70% acetone, respectively. Total content of proanthocyanidin in each fraction was determined by a vanillin assay. The fractions containing high concentrations of proanthocyanidin were pooled together and freeze-dried to obtain PRFR powder. The powder was resuspended in DMSO for total proanthocyanidin content determination using a vanillin assay and for further analysis.

### 4.3. Quantification of Proanthocyanidin Content

Total proanthocyanidin content in the PRFR was evaluated using a vanillin assay with slight modifications [35]. Briefly, PRFR (40 µL of 250, 500 and 1000 µg/mL) was mixed with 1% (*w*/*v*) vanillin in methanol (100 µL). Subsequently, 9 M sulfuric acid (100 µL of H_2_SO_4_) was added to the sample following incubation at 30 °C for 15 min. Proanthocyanidin content in the sample was quantified by spectrophotometry using an absorbance of 490 nm and compared with a standard of catechin. The amount of total proanthocyanidin content was expressed as milligram of catechin equivalents per gram of extract (mg CE/g extract).

### 4.4. Cell Cultures

Hepatocellular carcinoma, HepG2 cell line (HB-8065) was supplied from the ATCC (Manassas, VA, USA). HepG2 cells are derived from the liver tissue of a 15-year-old Caucasian male who had a well-differentiated hepatocellular carcinoma. The cell shows high expression of p21 and p27 with p53 wide type. The cells were cultured in DMEM supplemented with 10% (*v*/*v*) FBS, 100 U/mL penicillin and 100 μg/mL streptomycin. The cells were maintained in a 5% CO_2_ humidified incubator at 37 °C.

### 4.5. Cell Viability Assay

Cytotoxicity of PRFR in HepG2 cells was evaluated using the SRB assay, as previously described [36]. Briefly, the cells (1.5 × 10^4^ cells/well) were cultured in 96-well plates for 24 h. The cells were then treated with or without various concentrations (0–200 µg/mL) of PRFR in DMEM containing 10% FBS for 48 h. At the end of the treatment without removing the culture medium, cold 10% (*w*/*v*) trichloroacetic acid (TCA) solution (100 µL) was gently added to the plates followed by further incubation at 4 °C for 1 h. Subsequently, the medium was discarded, and the fixed cells were gently washed with slow running water. After air-drying at room temperature, the cells were stained with 0.057% (*w*/*v*) SRB solution (100 µL) for 30 min and then quickly rinsed four times with 1% (*v*/*v*) acetic acid. The pink color in the air-dried cells was solubilized using 10 mM tris base solution (pH 10.5). The absorbance at 510 nm was measured using a microplate reader.

### 4.6. Cell Cycle Assay

HepG2 cells were grown in 6-well plates (2 × 10^5^ cells/well) for 24 h and incubated with or without various concentrations of PRFR (0–40 µg/mL) for 48 h. The cell suspension was then prepared on ice and stained with propidium iodide (Guava Cell Cycle Reagent) for 30 min according to the Guava Cell Cycle Assay protocol. Cell cycle phase distribution was performed on a Guava PCA Instrument using CytoSoft Software (Merck-Millipore, Darmatadt, Germany).

### 4.7. Apoptosis Assay

HepG2 cells were grown in 6-well plates (2 × 10^5^ cells/well) for 24 h and incubated with or without various concentrations of PRFR (0–40 µg/mL) for 48 h. The cell suspension was then prepared and stained with annexin V-PE and 7-amino actinomycin D (7AAD) (Guava Cell Nexin Reagent) for 20 min according to the Guava Nexin Assay protocol. Apoptosis flow cytometry was carried out on a Guava PCA Instrument using CytoSoft Software.

### 4.8. Western Blot Analysis

HepG2 cells were grown in 35-mm culture dishes (1 × 10^6^ cells/dish) for 24 h and incubated with or without various concentrations of PRFR (0–25 µg/mL) for 24 h and 48 h. Proteins from HepG2 cells were extracted using RIPA buffer containing protease inhibitors. The concentration of the protein samples was then measured using Coomassie Plus™ Protein Assay Reagent and then subjected to 8–12% SDS-PAGE (20–40 µg/well). The proteins were transferred onto nitrocellulose membranes. The membranes were blocked with 5% skim milk in 0.05% TBS-tween. Subsequently, the membranes were probed with primary antibodies at 4 °C overnight in Can Get Signal^®^ Immunoreaction Enhancer Solution for caspase-3 and cleaved caspase-3, cyclin B1 and cdc25, and in 1% skim milk in 0.05% TBS-tween for PARP-1 and cleaved PARP-1, caspase-8 and cleaved caspase-8, survivin and p53. After being washed with 0.05% TBS-tween, the membranes were incubated with secondary antibodies-HRP conjugated anti-mouse or anti-rabbit IgG antibody in the same solution that was prepared for primary antibody conditions. The HRP signal was detected using an enhanced chemiluminescence (ECL) system. To confirm equal protein loading, each membrane was stripped and re-probed with the anti-β-actin antibody.

### 4.9. Statistical Analysis

All data are presented as mean ± standard deviation (S.D.) values. Statistical analysis was analyzed with Prism version 6.0 software (GraphPad software, San Diego, CA, USA) using one-way ANOVA with Dunnett’ s test. Statistical significance was determined at * *p* < 0.05, ** *p* < 0.01, *** *p* < 0.001 or **** *p* < 0.0001 versus control.

## 5. Conclusions

The PRFR derived from red rice germ and bran extract exhibits anti-carcinogenic properties in hepatocellular carcinoma, HepG2 cells in terms of anti-proliferation and apoptotic enhancements via survivin. PRFR halted cancer cells proliferation by inducing the cells to arrest at the G2/M phase of the cell cycle via the down-regulation of survival proteins, cyclin B1 and cdc25. In addition, PRFR triggered cancer cell death via the apoptosis pathway by enhancing active apoptotic proteins (i.e., cleaved PARP-1, cleaved caspase-8 and cleaved caspase-3) and lowering the anti-apoptotic protein survivin with tumor suppressor p53 independence. Taken together, PRFR may possess anti-cancer and cancer chemopreventive properties against liver cancer. Further research in animal studies is needed to better understand the potential impact of these findings.
